# Location, location, location: Fibrin, cells, and fibrinolytic factors in thrombi

**DOI:** 10.3389/fcvm.2022.1070502

**Published:** 2023-01-18

**Authors:** Anuj Narwal, Claire S. Whyte, Nicola J. Mutch

**Affiliations:** Aberdeen Cardiovascular and Diabetes Centre, School of Medicine, Medical Sciences and Nutrition, Institute of Medical Sciences, University of Aberdeen, Aberdeen, United Kingdom

**Keywords:** thrombus, fibrinolysis, platelets, plasminogen activators, fibrin

## Abstract

Thrombi are heterogenous in nature with composition and structure being dictated by the site of formation, initiating stimuli, shear stress, and cellular influences. Arterial thrombi are historically associated with high platelet content and more tightly packed fibrin, reflecting the shear stress in these vessels. In contrast, venous thrombi are generally erythrocyte and fibrin-rich with reduced platelet contribution. However, these conventional views on the composition of thrombi in divergent vascular beds have shifted in recent years, largely due to recent advances in thromboectomy and high-resolution imaging. Interestingly, the distribution of fibrinolytic proteins within thrombi is directly influenced by the cellular composition and vascular bed. This in turn influences the susceptibility of thrombi to proteolytic degradation. Our current knowledge of thrombus composition and its impact on resistance to thrombolytic therapy and success of thrombectomy is advancing, but nonetheless in its infancy. We require a deeper understanding of thrombus architecture and the downstream influence on fibrinolytic susceptibility. Ultimately, this will aid in a stratified and targeted approach to tailored antithrombotic strategies in patients with various thromboembolic diseases.

## Introduction

Thrombosis is the underlying pathology of major cardiovascular diseases, including myocardial infarction, ischemic stroke, and venous thromboembolism (VTE) which encompasses deep vein thrombosis and pulmonary embolism. Despite advances in diagnostics and novel antithrombotic drugs the mortality rate remains at 1 in 4 worldwide, creating a considerable burden on healthcare and society (1). In addition, thrombosis is a major cause of mortality in other disease, such as cancer, pathogenic infections, and autoimmune diseases (2–4). Hemodynamic forces and anatomic location significantly impact the formation, structure and stability of a thrombus within the vasculature. The resulting thrombi are heterogenous in nature, comprised of varying degrees of fibrin, platelets, erythrocytes, leukocytes, and neutrophil extracellular traps (NETs) (5). Thrombotic structures also vary within the arterial, venous, and microcirculation, at areas of turbulent flow, arising from atherosclerotic lesions, prosthetic devices or irregular vessel geometries and in different anatomic sites, such as the lung or ventricles of the heart (6). Analysis of thrombi has been hampered by availability of fresh samples, however, advancements in thrombectomy to remove thrombi from human blood vessels provided opportunities to examine the structure and composition of a thrombus (7). In addition, various *ex vivo* and *in vivo* models of thrombus formation and thrombolysis have provided useful tools to understand thrombus initiation in different vascular beds, the composition of various thrombus components, impact of shear and their downstream impact on fibrinolysis (8–12). Developing an understanding of thrombus composition, localization and abundance of fibrinolytic proteins in specific settings is crucial to personalize antithrombotic treatment strategies and develop novel drugs to target thrombosis.

## Thrombus initiation

**FIGURE 1:**
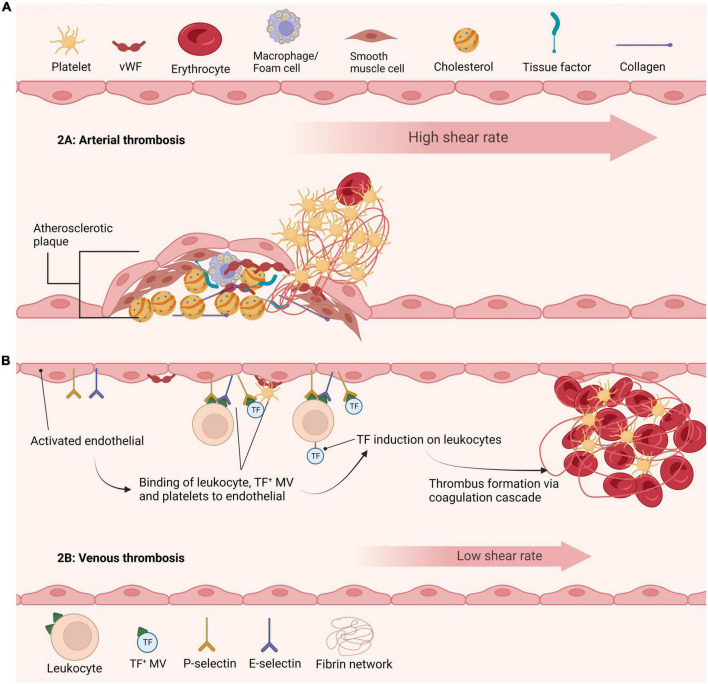
Current understanding of arterial and venous thrombi initiation. **(A)** Initiation of arterial thrombus formation is triggered by atherosclerotic plaque rupture. Exposure of collagen and tissue factor leads to recruitment and activation of platelets at the site of injury. Thrombi in arteries are formed under high shear stress and are rich in platelets. **(B)** Formation of venous thrombi is currently understood to be triggered by various mechanisms. The activated endothelium leads to recruitment and binding of cells and factors including leukocytes, tissue factor positive microvesicles and platelets. These agents further promote tissue factor recruitment ultimately leading to formation of a venous thrombus.

The trigger for thrombosis depends largely on the vascular bed (Figure 1). Nonetheless, an initial step is adherence of platelets to the vessel wall *via* various receptors, including the GPIb-IX-V/GPVI adheso-signaling complex thereby initiating platelet activation and aggregation (13). Activated platelets provide a catalytic aminophospholipid surface to assemble the prothrombinase complex, thereby catalyzing conversion of prothrombin to thrombin. These events elicit a conformation change in integrin αIIbβ3, allowing interaction with fibrinogen, which permits tethering of platelets to the forming fibrin network. Fibrinogen binding initiates outside-in signaling and promotes clot retraction, a process whereby activated platelets transduce contractile forces to the fibrin network augmenting clot density and decreasing clot size. Clot retraction is important for clot stability and maintaining blood vessel patency. Interestingly, a recent study found a direct link between endogenous fibrinolysis and clot retraction, suggesting that these processes are inextricably linked *in vivo* (14). Additional platelet receptors for fibrin have been proposed, including GPVI (15), which can directly instigate platelet activation and drive thrombus propagation (16).

Differential agonist distribution in the evolving platelet mass results in phenotypically different subpopulations of platelets (17–20) within the microenvironment of the thrombus. Phosphatidylserine (PS)-negative (aggregating platelets) have a spread morphology, avidly bind fibrinogen, *via* activated αIIbβ3, and generate fibrin on their surface (21). PS-exposing platelets (procoagulant) bind the prothrombinase complex and exhibit a characteristic balloon shape with prolonged spikes in cytosolic Ca^2+^; these platelets lack activation of integrin αIIbβ3 (20–22). Work from our group (10, 23, 24) and others (25–28) has shown that PS is concentrated in the “cap” or “body” of these platelets along with key hemostatic proteins.

Thrombin generation on the activated platelets amplifies fibrin formation, thereby providing structural support, mechanical stability and integrity to the thrombus. Fibrin structure is altered by multiple parameters, and is largely dictated by fibrinogen and thrombin concentration (29). Low thrombin concentrations generate thick fibrin fibers, loosely woven, and permeable fibrin clots leading to hyperfibrinolysis. In contrast, high thrombin concentrations give rise to clots comprising of a dense network of thin fibrin fibers associated with hypofibrinolysis (30). Various studies have shown that compact fibrin structures, with highly branched networks are associated with pathophysiological diseases such as, coronary artery disease, ischemic stroke and pulmonary embolism [reviewed by Undas and Ariens (31)]. Fibrin fibers orient in the direction of flow, with increased alignment as shear stress is magnified (32). Interestingly, recent studies indicate that fibrin forms a protective biofilm over the external area of a thrombus, as a protection against invading pathogens (33).

## Impact of thrombus location and shear stress

### Arterial thrombosis

Arterial thrombosis is typically triggered by rupture of an atherosclerotic plaque, permitting contact of highly prothrombotic material, rich in tissue factor and lipids, with plasma thereby prompting platelet activation and coagulation (Figure 1A). Structural analysis of coronary arterial thrombi revealed they are comprised of fibrin (43% of thrombus volume) and platelets (31%) (34). Fibrin was tightly packed, perhaps not surprising given the high shear stress (1,000–1,500 s^–1^), and arranged in bundles, possibly reflecting the lateral association of fibers due to increased compression exerted by platelet contraction (35). Interestingly, a small number of compressed erythrocytes, termed polyhedrocytes, were evident and the remainder of thrombus volume was occupied by microvesicles and leukocytes (34). Thrombi from ST-segment-elevation myocardial infarction (STEMI) patients, were again largely composed of fibrin with increased erythrocyte to platelet ratio than reported in coronary artery thrombi (36). Intriguingly, in primary coronary intervention fibrin content correlated with plasminogen activator inhibitor-1 (PAI-1) and P-selectin, indicative of a role for platelets in driving fibrin formation (36).

Interestingly, thromboemboli retrieved from the middle cerebral artery or intracranial carotid artery of patients with acute ischemic stroke revealed significant heterogeneity, but again platelet-fibrin areas were dominant, interspersed with areas of nucleated cells and erythrocytes (37). More recent studies unveil heterogenous areas, comprised of erythrocyte-rich and fibrin poor areas and platelet- and fibrin-rich areas (38). A recent elegant study of thrombi from acute ischemic stroke, using scanning electron microscopy and immunohistochemistry, revealed an outer shell, comprised of densely packed fibrin, von Willebrand factor, and aggregated platelets (39). Parameters affecting thrombus growth shift at the point of occlusion when shear stress decreases due to diversion of the blood. Nonetheless, microfluidic modeling of occlusive thrombus formation that permits pressure release demonstrated that despite the variations in shear stress fibrin accumulation under arterial rates was still reduced in comparison to the venous circulation (40).

### Venous thrombosis

The mechanisms underpinning development of venous thrombosis are still debatable, with a call for action and prioritization of funding in this area (41). The concepts of Virchow’s triangle, including changes in blood composition, reduction in blood flow, and changes to the vascular endothelium are considered key drivers, but further work is required to tease out precise mechanisms. Genetic and acquired risk factors augment the risk of venous thrombosis [reviewed by Wolberg et al. (42)]. A pivotal study by von-Bruhl et al. (43) demonstrated that initiating events of venous thrombosis *in vivo* involve crosstalk between platelets, monocytes and neutrophils (Figure 1B). They elegantly demonstrated that neutropenia, genetic ablation of FXII, or disintegration of NETs individually confer protection against deep vein thrombosis (DVT) *in vivo* (43).

Erythrocytes comprise nearly 60% of the volume of venous thrombi with fibrin fibers accounting for about 30% (34). Polyhedrocytes were also found in venous thrombi, with around 5% of thrombus volume composed of echinocytes (34). These “thorny” erythrocytes are indicative of oxidative stress and perhaps cellular aging within the thrombus environment. Leukocytes and microvesicles were detected but were less abundant (34). The endothelial contribution in venous thrombosis is vital, as it captures leukocytes, tissue factor-positive microvesicles and platelets (Figure 1B). The composition of pulmonary emboli (PE) largely mirrored that of venous thrombi, with polyhedrocytes accounting for majority of the thrombus volume (34). A recent report indicates that PE thrombi are generally “earlier” stage in terms of composition with a higher erythrocyte component (44). Intriguingly, within venous thrombi fibrin fibers were largely evident as individual fibers rather than bundles, perhaps reflecting a decrease in mechanical stability, and accounting for their tendency to readily embolize.

Severe COVID-19 disease is associated with an increased risk of thrombosis (45), both systemically and locally within the pulmonary vasculature (46). Studies indicate that PE derived from critically ill COVID-19 patients differ significantly from non-COVID PE (47). Thrombi were located directly within opacitated lung segments, indicative of *in situ* thrombogenesis (48). An increased rate of *in situ* PE in COVID-19 may suggest that leukocytes drive thrombogenesis. Indeed, an *in vivo* model of DVT has revealed significant fibrin deposition in rats with normal neutrophil counts which is attenuated in neutropenia (49). Further research is required to directly compare the structural composition of PE formed *in situ* vs. those that embolize to the pulmonary vasculature which will aid understanding of underlying mechanisms and personalize diagnosis and care.

## Mechanistic contributions of cells to thrombus composition

The mechanistic contributions of various circulating cells, including erythrocytes and inflammatory cells, to thrombosis is currently the subject of intense scientific scrutiny. Many avenues of interplay between hemostatic factors and cells or cell-cell interactions have and continue to be uncovered, some of these are highlighted below.

### Erythrocytes

Erythrocytes were long considered to be innocent bystanders in thrombi but are now considered to play a more significant role than previously thought [reviewed by Byrnes and Wolberg (50)]. Erythrocytes express the Fas ligand, FasL and the death receptor, FasR (51). Activation of FasR induces loss of asymmetry and integrity of the phospholipid bilayer thus exposing aminophospholipids. This provides an “eat-me” signal to remove older erythrocytes from the circulation, however, these aminophospholids can also assemble the prothrombinase complex leading to thrombin generation. ADP-activated platelets express FasL on their membrane which interacts with FasR on erythrocytes augmenting aminophospholipid exposure (52). To date this unique cell-cell interaction has only been demonstrated *in vitro*, however, it provides a novel mechanism in which erythrocytes can promote thrombus formation.

Erythrocyte aggregation influences blood flow and is a cardiovascular risk factor. It was hypothesized that fibrinogen and other plasma proteins induced erythrocyte aggregation *via* non-specific binding. However, Carvalho et al. (53) demonstrated a unique interaction between fibrinogen and an unknown receptor on erythrocytes using atomic force microscopy. A patient with Glanzmann thrombastenia, a hereditary bleeding disorder caused by deficiency of integrin αIIbβ3, showed defective binding of fibrinogen to erythrocytes. Similarly, the αIIbβ3 inhibitor, eftifibatide, attenuated binding of fibrinogen to erythrocytes, albeit to a lesser degree than on platelets. Interestingly, mice carrying a homozygous mutation for γ390-396 in fibrinogen showed a 50% reduction in thrombus weight, due to reduced erythrocyte volume (54). This effect was mediated *via* factor XIII activation and crosslinking (54). The group later showed this was dependent on the presence of plasma FXIII (55) and that retention of erythrocytes in clots is mediated *via* fibrin α-chain cross-linking (56). As discussed, erythrocytes accrued within the clot are frequently observed as polyhedrocytes rather than their native bioconcave state (34, 57). It is the process of clot contraction, mediated by platelets that generates the necessary force to compress and alter the rigidity of erythrocytes into these tightly packed arrays of polyhedrocytes (58, 59). These lines of evidence indicate that erythrocytes play an active role in the size and structural integrity of pathophysiological thrombi.

### Leukocytes

The intrinsic link between the innate immune system and coagulation is now firmly established (60). Fibrin(ogen) binds to the integrin α_M_β_2_ which is crucial for leukocyte function and innate immunity *in vivo* (61). Platelet-leukocyte aggregates, mediated *via* interaction of platelet P-selectin and GPIbα with neutrophil P-selectin glycoprotein ligand-1 and αMβ2 integrin, respectively, are common features in thromboinflammatory disorders [reviewed by Swystun and Liaw (62)]. This cell-cell interaction induces a hypercoaguable state inciting platelet activation, binding of coagulation factors and adhesive proteins such as von Willebrand factor (vWF).

Monocytes harbor the largest circulating pool of tissue factor, which is principally in a quiescent state but can be exposed and decrypted in response to various inflammatory stimuli. Activated monocytes shed microvesicles that carry tissue factor, expose PS, and other procoagulant factors (63). It is well known that monocytes house a large pool of intracellular factor XIII-A (64). Our laboratory has recently shown that monocytes externalize factor XIII-A in response to inflammatory stimuli which stabilizes thrombi in a transglutaminase-dependent manner (65). Monocytes are also the largest circulating pool of the fibrinolytic inhibitor, PAI-2 which is upregulated in response to thrombin and LPS stimulus (66). Interestingly, this serpin is considered to be largely intracellular in nature, but can down-regulate uPA and is cross-linked to fibrin (67). PAI-2 is also found in extracts of human arterial and venous thrombi suggesting secretion from monocytes in response to various stimuli (67). Mice deficient in PAI-2 exhibit superior venous thrombus resolution due to inflammatory and uPA-mediated mechanism (68). Conversely, reports indicate that monocytes recruitment into the thrombi is important for resolution, which is largely uPA-mediated (69). Clearly there is a strong need to understand the nuances by which immune cells function to explain existing controversies in the literature and their role in governing thrombus stability.

Neutrophils accumulate at sites of injury acting to limit invading pathogens. Brinkmann et al. described the extrusion of neutrophil nuclear and cytoplasmic content forming NETs in the cell death process of NETosis (70). These web-like structures are formed in response to inflammatory stimuli, microbial invasion and are composed of histones, DNA strands and granular proteins including neutrophil elastase (70). NETs have been detected in both venous (71) and arterial thrombi (72–74). NETs contribute to thrombus formation through multiple mechanisms, including the release of neutrophil elastase and cathepsin G, as well as externalization of nucleosomes (75). NETs expose tissue factor and protein disulfide isomerase, an enzyme responsible for activating blood cell derived tissue factor (43, 76, 77) thereby driving coagulation. NETosis is promoted under high shear conditions (78) independent of thrombin and fibrin generation (79). Indeed, fibrin limits NET formation and tPA facilitates shear-induced NET formation (78). NETs promote platelet adhesion, activation and aggregation (80) and citrullinated histone H3 (CitH3) are detected in close proximity to vWF within fibrin-rich areas of thrombi (81). Conversely, platelets contribute to the formation of NETs through lipopolysaccharide binding of Toll like receptor 4 (TLR4) (82).

## Localization of fibrinolytic activity

**FIGURE 2:**
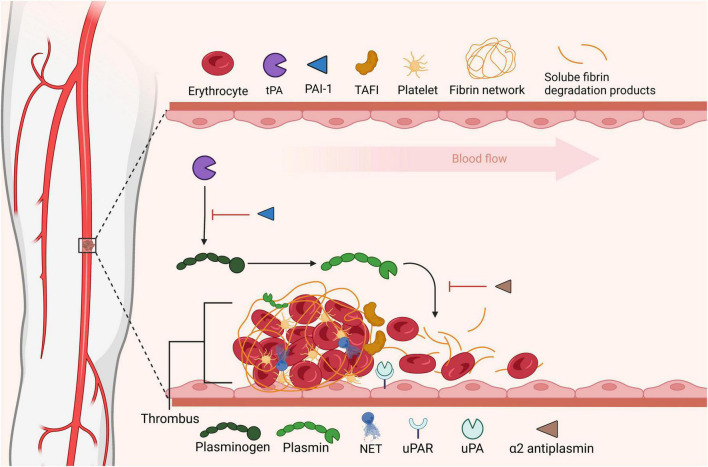
Fibrinolytic dissolution of thrombi. Fibrinolysis is initiated by plasminogen activators that convert circulating plasminogen to its active form plasmin promoting degradation of fibrin. Soluble fibrin degradation products can be cleared from the circulation. Fibrinolysis is regulated at the level of plasminogen activation, *via* plasminogen activator inhibitor-1 (PAI-1) or by direct inhibition of plasmin by α2-antiplasmin (α2AP). Thrombin activable fibrinolysis inhibitor (TAFI) impedes fibrinolysis by removing C-terminal residues from fibrin, these lysine residues are vital for plasminogen binding to fibrin. In this figure was adapted from “Tissue Plasminogen Activator Activity at Ischemic Region in the Brain,” by BioRender.com (2022). Retrieved from https://app.biorender.com/biorender-templates.

The fibrinolytic system is nature’s endogenous system programmed to dissolve intravascular clots and counteract the opposing coagulation system (Figure 2). Plasmin, the key proteolytic enzyme, is formed *via* cleavage of circulating plasminogen through the action of plasminogen activators, primarily tissue-type PA (tPA) and uPA. Endothelial cells (83), neurons (84) and hepatocytes (85) express and secrete tPA, with recent evidence suggested that hepatocyte-derived tPA contributes to basal circulating levels of tPA. In contrast, uPA is largely expressed by migratory and inflammatory cells (86). The system is governed by several inhibitors, including α2-antiplasmin and PAI-1 and PAI-2. Activated thrombin activatable fibrinolysis inhibitor (TAFIa; *CPB2*) down-regulates fibrinolysis, *via* removal of C-terminal lysine residues from partially degraded fibrin, thereby attenuating binding of plasminogen and tPA. In thrombosis the fibrinolytic balance is disturbed, favoring fibrin formation and persistence, which can be partially attributed to the cellular composition of thrombi and their relative contributions to the system.

### Profibrinolytic activity

Accumulation of tPA and plasminogen is observed in the head of Chandler model thrombi, directly aligning with localization of fibrinolytic activity (12). This observation is unexpected given the knowledge that the head is rich in platelets and leukocytes, while the tail is fibrin-rich (8). Our previous work had established that the cellular-rich head is rich in active uPA, which is largely leukocyte in origin (9). Within this microenvironment proteolytic activity of the plasminogen activators is largely protected from inhibition by PAI-1 (12), contrasting the situation in plasma where tPA is largely found in complex with PAI-1 (87). Elevation in endogenous tPA during thrombus formation increases retention within thrombi (12), indicating that thrombus resolution is dictated by the levels of activators present during formation. However, there is evidence of infiltration of monocyte/macrophages and neutrophils into forming thrombi (43, 69, 81, 88). Accumulation of plasminogen in thrombi, formed under shear stress, has been depicted by our laboratory (10), plasminogen was demonstrated to be primarily localized in the thrombus core directly on fibrin and on the surface of PS-exposing platelets, *via* both fibrin dependent and independent processes (10). Consistent with this, plasminogen accumulated in the thrombus core with PS-exposing platelets in an *in vivo* laser-injury model and this process was enhanced by endogenous plasmin activity (89). Similarly, plasminogen accumulates in fibrin-rich areas on preformed thrombi formed under high shear rates (90). Model thrombi formed using non-anticoagulated blood under high shear rates show elevated levels of PAI-1, whilst both tPA and plasminogen were reduced, resulting in slower rates of fibrinolysis compared to that in thrombi formed at low shear (12). Indeed, cells that are incorporated into thrombi harbor many receptors for plasminogen, largely utilizing C-terminal lysines (91). Plg-R_KT_ was identified in 2010 as the first receptor for plasminogen to be synthesized with a C-terminal lysine (92). Plg-R_KT_ demonstrates affinity for tPA and is known to co-localize with uPAR on monocytes and macrophages (92). We have subsequently identified Plg-R_KT_ on platelets and found that it is directly responsible for anchoring plasminogen to the activated platelet membrane (93). Interestingly, while platelets do not express uPAR, we have shown that the platelet membrane stimulates reciprocal activation of scuPA and plasminogen to their active forms (94), thereby highlighting the importance of cellular surfaces in regulating profibrinolytic activity. Intriguingly, plasminogen bound to fibrin, platelets or extracellular matrix proteins can be proteolytically activated by uPA adhered to monocytes or microvesicles (95). Binding of soluble Glu-plasminogen to cell surfaces enhances its activation [reviewed in Miles and Parmer (96)] induces a conformational change distinct from that of Lys-plasminogen (97). These studies and others highlight the importance of the cell membrane in supplying fibrinolytic proteins and catalyzing plasminogen activation.

### Antifibrinolytic activity

Platelets are the major pool of circulating PAI-1 (98). Degranulation following platelet activation gives rise to release of platelet-derived PAI-1 into the local milieu (99, 100). Our laboratory has recently showing that functional PAI-1 is retained on the activated platelet membrane and on associated fibrin (101), providing a local pool of serpin within the thrombus. Platelets also contain an abundance of other fibrinolytic inhibitors such as, alpha2-antiplasmin (102, 103) protease nexin I (PN-1) (104–106), C1-inhibitor (107, 108) and TAFI (109). These inhibitors are also secreted following activation and contribute to antifibrinolytic capacity. PN-1 from platelets downregulates the plasmin generating ability of fibrin-bound tPA and the activity of fibrin-bound plasmin and inhibits uPA (106, 110). However, as noted tPA, uPA and plasminogen are largely protected from inhibition if fibrin- or cell-bound. Activated TAFI (TAFIa) has been shown to limit plasminogen and tPA accumulation on the platelet surface and movement within plasma clots (111, 112). Platelets are also a rich-source of factor XIII-A (113–115) which is known to be externalized upon activation and can participate in thrombus stabilization *via* crosslinking of a_2_antiplasmin into the forming thrombus (24). Targeting of activated platelets is therefore an attractive therapeutic strategy. Single-chain antibodies to the platelet integrin α_IIb_β_3_ fused to scuPA have shown promise in a mouse ischemic stroke model (116).

Platelet-mediated clot retraction is reportedly resistant to external fibrinolysis, however, is vulnerable to endogenous fibrinolysis (14). Interestingly, the internal rate of fibrinolysis is enhanced by clot retraction, whilst the external rate of fibrinolysis is impeded thereby suggesting differences in the fibrin susceptibility due to structural rearrangements during this process (117). Thrombi containing erythrocytes formed *in vitro* are more resistant to plasmin-mediated fibrinolysis despite the thrombi being composed of thinner fibers and a less dense fibrin network (118). However, thrombi obtained from stroke patients by endovascular thrombectomy that were more responsive to intravenous thrombolysis were found to be more erythrocyte-rich (119). Higher erythrocyte count has been associated with shorter intervention times, lower thrombolysis resistance and incidences of embolism and successful recanalization (120–122). The presence of higher white blood cell counts, NETs and vWF have been linked to reduced rates of recanalization (123). Clearly, there is a need for deeper research in this area to deepen our understanding of this area and iron out discrepancies in the current literature.

In addition to “conventional” fibrinolytic factors additional modifiers of thrombus stability have been identified. The impact of NETs, and specifically the DNA composition of thrombi, in limiting fibrinolysis has recently garnered attention. Histones alter fibrin fiber thickness and are crosslinked *via* factor XIIIa into the network which downregulates fibrinolysis (124). There is significant interest in inclusion of a DNase enzyme, as an adjunct to Alteplase (Actilyse^®^) in thrombolytic therapy. DNase1, an endonuclease that facilitates chromatin breakdown, has been shown to reduce NET formation and considerably limit DVT growth in mice (43). Additionally, DNase accelerates the rate of *ex vivo* thrombolysis of coronary and acute ischemic stroke thrombi (72, 73). The presence of large vWF multimers formed under high shear conditions also confer thrombolytic resistance, due to resistance to ADAMTS13, which cleaves vWF and tPA (125). Thrombotic thrombocytopenic purpura (TTP) is caused by ADAMTS13 deficiency leading to ultra-large vWF multimers. Targeted plasmin-mediated degradation of vWF polymers using fusion of a nanobody targeting vWF with the protease domain of uPA has recently shown promise as a treatment for TTP (126).

## Conclusion

The recent advances in novel *ex vivo* models combined with *in vivo* animal models and developments in thrombectomy have significantly improved our understanding of the complex thrombus environment. This in turn gives significant insight into the susceptibility of thrombi to lysis and the factors which govern these processes. Understanding the impact of location, shear stress and vessel geometries on the cellular content and fibrin network is essential for the development of targeted and personalized approaches to treat thrombotic complications.

## Author contributions

AN wrote the manuscript and designed the figures. CW and NM conceptualized, wrote, edited and reviewed the manuscript and figures. All authors contributed to the article and approved the submitted version.
